# Vision-based diagnostic gain of ChatGPT-5 and gemini 2.5 pro compared with human experts in oral lesion assessment

**DOI:** 10.1038/s41598-025-28862-1

**Published:** 2025-12-05

**Authors:** Fatma E. A. Hassanein, Radwa R. Hussein, Hadeel Gamal Almalahy, Susan Sarhan, Yousra Ahmed, Asmaa Abou-Bakr

**Affiliations:** 1 Oral Medicine, Periodontology, and Oral Diagnosis, Faculty of Dentistry, King Salman International University, El-Tor, Egypt; 2 Oral Medicine, Periodontology, Ain Shams University in Egypt, Cairo, Egypt; 3 Prosthodontics Dentistry, Faculty of Dentistry, King Salman International University, El-Tor, Egypt; 4 Oral Medicine and Periodontology, Faculty of Dentistry, Galala University, Suez, Egypt

**Keywords:** Artificial intelligence, Oral medicine, Oral lesions, Diagnosis, Large language model, ChatGPT 5, Gemini 2.5 pro, Computational biology and bioinformatics, Diseases, Health care, Medical research

## Abstract

The diagnostic potential of multimodal large language models (LLMs) in oral medicine remains underexplored, particularly in real-world clinical contexts. This study introduces *Vision-Based Diagnostic Gain (VWDG)* as a novel metric to quantify the incremental diagnostic value of incorporating images into AI-assisted diagnosis of oral lesions. We conducted a prospective, biopsy-validated, case-matched study including 200 oral lesion cases with clinical photographs and radiographs of variable quality. ChatGPT-5 and Gemini 2.5 Pro were evaluated against board-certified oral medicine experts. Each case was presented under two conditions: text-only and multimodal (text plus images). Diagnostic accuracy was measured across Top-1, Top-3, and Top-5 differentials. *VWDG* was defined as the absolute and relative improvement in diagnostic accuracy between multimodal and text-only conditions. Cochran’s Q and paired McNemar tests with effect sizes quantified differences across models and conditions, with analyses stratified by lesion type and diagnostic difficulty Both models demonstrated strong baseline diagnostic accuracy, but their performance diverged with image integration. ChatGPT-5 achieved significant VWDG across thresholds—Top-1 gain + 19% points, Top-3 gain + 18 pp, and Top-5 gain + 14 pp (all *p* < 0.001). In contrast, Gemini 2.5 Pro showed negligible or even negative gain (0 pp at Top-1/Top-3; − 2 pp at Top-5). Stratified analyses confirmed that ChatGPT-5 benefited most from visual input in malignant and diagnostically difficult cases, whereas Gemini’s strength remained in text-dominant contexts. Human experts consistently outperformed both models in simple and benign presentations. By introducing and applying VWDG, this study provides the first expert-anchored, head-to-head evaluation of next-generation multimodal LLMs in oral medicine. ChatGPT-5 functions as a *visual synergist*, Gemini as a *textual expert*, and their complementary strengths suggest a cooperative human–AI diagnostic paradigm. VWDG offers a clinically meaningful framework for benchmarking AI models and guiding safe, context-aware integration into practice.

## Introduction

Artificial Intelligence (AI) has emerged as a revolutionary tool in several healthcare domains, enhancing patient management, treatment planning, and illness diagnosis^1^. Using machine learning (ML) to gather patient data to enhance clinical judgment and ultimately patient outcomes is a significant use of AI in healthcare^2^. Additional advantages of AI include enabling healthcare workers to communicate with their colleagues around the world^3^, cutting down on time-consuming, mundane tasks^4^, putting in place an appropriate, individualized patient management system, and maybe providing healthcare remotely^5^.

In oral medicine specifically, prompt and accurate identification of oral diseases including oral cancer, potentially malignant disorders, and infectious lesions—is essential for favorable prognosis and reduced morbidity. Yet reliable diagnosis often depends on specialist expertise, which is limited in many low-resource settings, creating major barriers to timely care.

Despite significant progress in radiology and dermatology, the uptake of AI in oral-medicine diagnostics has lagged. Most published work remains laboratory-bound, relying on curated, high-quality image repositories rather than the heterogeneous material clinicians face daily^6,7^. This gap between bench-top benchmarks and chair-side decision-making has slowed translation into practice. A recent study by Hassanein et al. (2025) began to bridge this divide by benchmarking ChatGPT-4o and DeepSeek-3 on 80 image-integrated clinical vignettes, showing that lesion type and diagnostic difficulty strongly influenced accuracy. However, even in this multimodal setting, experts maintained a Top-1 advantage, underscoring that LLMs have yet to match human diagnostic consistency.

The need for reliable diagnostic support is amplified by well-documented shortcomings in clinical accuracy. Specialist care is scarce in many regions, with patients often consulting multiple providers and facing referral delays exceeding one year^1^. Even in specialized clinics, misdiagnosis rates are concerning: clinical errors have been reported in 31–43% of cases^8,9^, while clinicopathologic agreement hovers around 60%, and sensitivity for malignancy may be as low as 45%^10^. These figures highlight not only the difficulty of oral lesion diagnosis but also the urgent need for accessible adjunctive tools to improve accuracy, consistency, and timeliness of care.

While AI has already demonstrated substantial benefits in medical image analysis—including oral lesions, radiology, and dermatology—with clear gains in efficiency and diagnostic precision^11–16^, lesion type and imaging modality remain critical determinants of model performance. In oral medicine, where both text-based narratives and visual morphology are central, unimodal AI systems remain insufficient. Most prior evaluations of LLMs have focused on text-only prompts, reported modest accuracies (e.g., 64% for GPT-4o and 35% for Gemini on potentially malignant lesions)^17^, and tested single systems in isolation. Even when limited imaging inputs were incorporated, diagnostic performance plateaued around 78% in rare case scenarios^18^. Thus, the real-world diagnostic potential of multimodal LLMs remains largely unexplored.

This study addresses a critical translational gap by introducing and applying Vision-Based Diagnostic Gain (VWDG) as a metric to evaluate AI diagnostic utility in real-world practice. Unlike earlier work limited to curated datasets, we assessed performance on variable-quality clinical photographs and radiographs that reflect daily clinical conditions. Two advanced multimodal large language models (ChatGPT-5 and Gemini Pro 2.5) were benchmarked against board-certified oral medicine experts in a biopsy-validated, case-matched design. Stratified analyses by lesion type and diagnostic difficulty, coupled with robust paired statistical methods, enabled the first large-scale, expert-anchored, head-to-head comparison of next-generation multimodal LLMs in oral medicine. By quantifying the incremental diagnostic value of visual prompting through VWDG, this study highlights the scenarios where AI can function as a safe and effective adjunct, advancing evaluation beyond accuracy metrics toward clinically meaningful utility.

## Methods

### Study design and setting

This was a prospective, paired diagnostic accuracy study conducted across university oral medicine clinics in Egypt. The study compared two state-of-the-art large language models (LLMs) ChatGPT-5 (OpenAI) and Gemini Pro 2.5 (Google) with board-certified oral medicine experts in generating differential diagnoses for real patient cases. A matched-pairs design was used, whereby each case was assessed by both AI models and the human experts, enabling within-case performance comparisons.

### Ethical considerations

All participants in this study provided informed oral and written consent following the institutional and national research committee, including the 1964 Helsinki Declaration and its later amendments. Written informed consent was also obtained from all patients before their participation. Permission was secured to use their de-identified clinical data and photographic images for research and scientific publication. Patient confidentiality was fully maintained throughout the study, and no names or private information were disclosed or published. The study received approval from the ethical committee of the Faculty of Dentistry at Ain Shams University, and it was assigned the number FDAs-Rec ID032209.

### Case selection & sample-size justification

Adult patients (≥ 18 years) presenting with clinically evident oral lesions that required biopsy were prospectively recruited from Ain Shams University, King Salman International University, and Galala University clinics. Exclusion criteria were: prior surgical or radiotherapy treatment of the lesion, incomplete clinical records, or inadequate photographic quality, or lack of histopathological studies. Sample size was determined based on effect sizes reported by Hassanein et al. (2025), which observed up to a 35% difference in Top-1 diagnostic accuracy between AI models and human experts^19^. Assuming a 20% absolute difference in Top-1 accuracy (e.g., 60% vs. 80%), with α = 0.05 and 80% power using McNemar’s test, a minimum of 80 cases was required per comparison. To accommodate subgroup analyses and multiple model comparisons, a total of 160 evaluable cases was targeted. Anticipating a 20% exclusion rate, 200 vignettes were initially screened.

### Case development

For each participant, standardized clinical vignettes were constructed, incorporating: (1) demographic details, (2) relevant medical and dental history, (3) lesion description (site, size, appearance, and symptoms), and (4) high-resolution intraoral photographs. Radiographs were included when clinically indicated. All cases were independently validated by two oral medicine specialists prior to inclusion to ensure representativeness.

### Reference standard

The histopathological diagnosis served as the gold standard reference. All biopsies were assessed by board-certified oral pathologists blinded to AI and expert assessments.

### Index tests (AI Models)

Two LLMs were evaluated:

- *ChatGPT-5* (OpenAI, August 2025 release).
- *Gemini Pro 2.5* (Google, June 2025).

Both models were accessed in their production versions without fine-tuning, to reflect real-world conditions. Each case vignette (text + photo) was presented under two conditions:

1. *Text-only prompt*: “According to the following clinical case, please provide five most likely diagnoses ranked by probability”.
2. *Text + image prompt*: same prompt with clinical photographs (and radiographs where available).

Sessions were isolated, with history cleared after each case. A warm-up case (not included in the analysis) was used to align model behavior.

### Comparator (Human Experts)

Two board-certified oral medicine consultants (> 10 years’ experience) independently reviewed all 200 cases. Each provided a ranked Top-5 differential diagnosis list based solely on the vignette and photographs. Human experts received the complete multimodal information (text and images) for all cases, reflecting real-world diagnostic workflows in oral medicine where visual and contextual cues are interpreted jointly. In contrast, the AI models (ChatGPT-5 and Gemini 2.5 Pro) were tested under both *text-only* and *text + image* conditions to quantify their Vision-Based Diagnostic Gain (VBDG). The textual and visual content presented to experts was identical to that used for the AI models, ensuring informational equivalence while varying input modality only for the LLMs. Discrepancies were resolved through consensus discussion, yielding a final expert comparator list.

### Stratification variables

To explore heterogeneity of performance, each case was prospectively stratified by:

- *Lesion type*: Cases were categorized as inflammatory/infectious, reactive, benign, or malignant, following histopathological criteria defined by the World Health Organization (WHO) Classification of Head and Neck Tumors, 5th Edition (2022)^20^. The inflammatory/infectious group included oral candidiasis, mucositis, and nonspecific chronic inflammatory lesions; the reactive group comprised pyogenic granuloma, inflammatory fibrous hyperplasia, traumatic ulcer, and oral lichen planus; the benign group encompassed fibroma, squamous papilloma, lipoma, and benign salivary gland adenoma; and the malignant group included oral squamous cell carcinoma, verrucous carcinoma, and mucoepidermoid carcinoma. All classifications were validated independently by two oral medicine specialists and confirmed by blinded histopathological examination to ensure diagnostic consistency and reproducibility.
- *Diagnostic difficulty*: Diagnostic difficulty was determined based on the overall clinical complexity across lesion categories. Cases were classified as low when the lesions were common, well-defined, and exhibited pathognomonic features with minimal overlap; moderate when diagnostic interpretation required integration of clinical history due to overlapping or atypical features; and high when the lesions were rare, highly atypical, or showed substantial ambiguity and confounding features. Inter-rater reliability was confirmed using Fleiss’ κ, and any discrepancies were resolved by consensus. These criteria were adapted from standard oral medicine and pathology references^21,22^ and a previously published diagnostic-ambiguity framework^19^.

### Scoring protocol

For each model and expert list, diagnostic accuracy was measured as:

- *Top-1 accuracy*: correct diagnosis ranked first.
- *Top-3 accuracy*: correct diagnosis listed among the top three.
- *Top-5 accuracy*: correct diagnosis listed among the top five.

Scores were binary (1 = correct, 0 = incorrect).

### Vision-based diagnostic gain analysis

The incremental value of visual input was quantified by comparing accuracy under text-only versus text + image conditions. Three metrics were calculated:

- Absolute gain (image – text accuracy).
- Relative gain (image/text ratio).
- Error reduction (%) = (Error_text – Error_image)/Error_text ×100.

Comparisons were performed using McNemar’s test, Cohen’s h, and Wald-based statistics. Subgroup analyses assessed vision-based gains by lesion type and diagnostic difficulty.

### Statistical analysis

All analyses were conducted in Stata 18.0 and R 4.3.3 with α = 0.05 (two-tailed). Diagnostic accuracy across AI models was compared at Top-1, Top-3, and Top-5 ranks using Cochran’s Q tests (df = 3) and Bonferroni-adjusted McNemar pairwise tests. Vision-Based Diagnostic Gain (VWDG) was calculated as absolute gain, relative gain, and error reduction, with between-model differences assessed by Wald tests and standardized using Cohen’s h. Stratified analyses by lesion type and case difficulty were performed with paired McNemar tests and effect sizes. Within-case logistic regression was used to estimate odds ratios (ORs, 95% CI) for incremental image effects, applying Haldane–Anscombe correction and exact McNemar tests when discordant counts were ≤ 25.

## Results

**Fig. 1:**
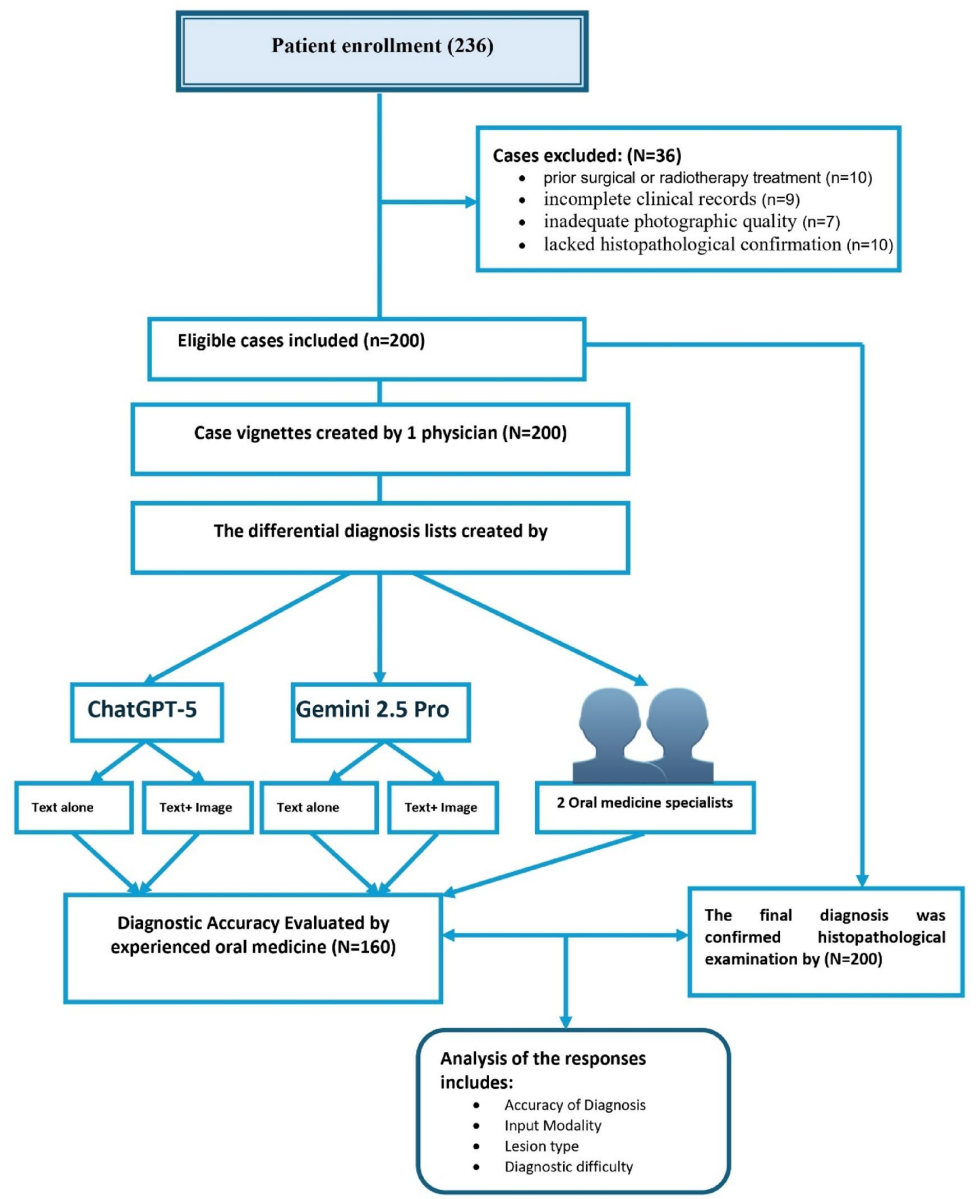
Study design.

A total of 236 patients with clinically evident oral lesions were prospectively enrolled across participating oral medicine clinics. Of these, 36 cases were excluded for the following reasons: 10 had a history of prior surgical or radiotherapy treatment of the lesion, 9 had incomplete clinical records, 7 presented with inadequate photographic quality, and 10 lacked histopathological confirmation. The final analytic cohort comprised 200 biopsy-confirmed cases, each represented by a standardized vignette with clinical photographs (and radiographs where available), which were used for diagnostic accuracy comparisons between ChatGPT-5, Gemini Pro 2.5, and oral medicine experts (Fig. 1).

### Overall diagnostic accuracy

**Fig. 2:**
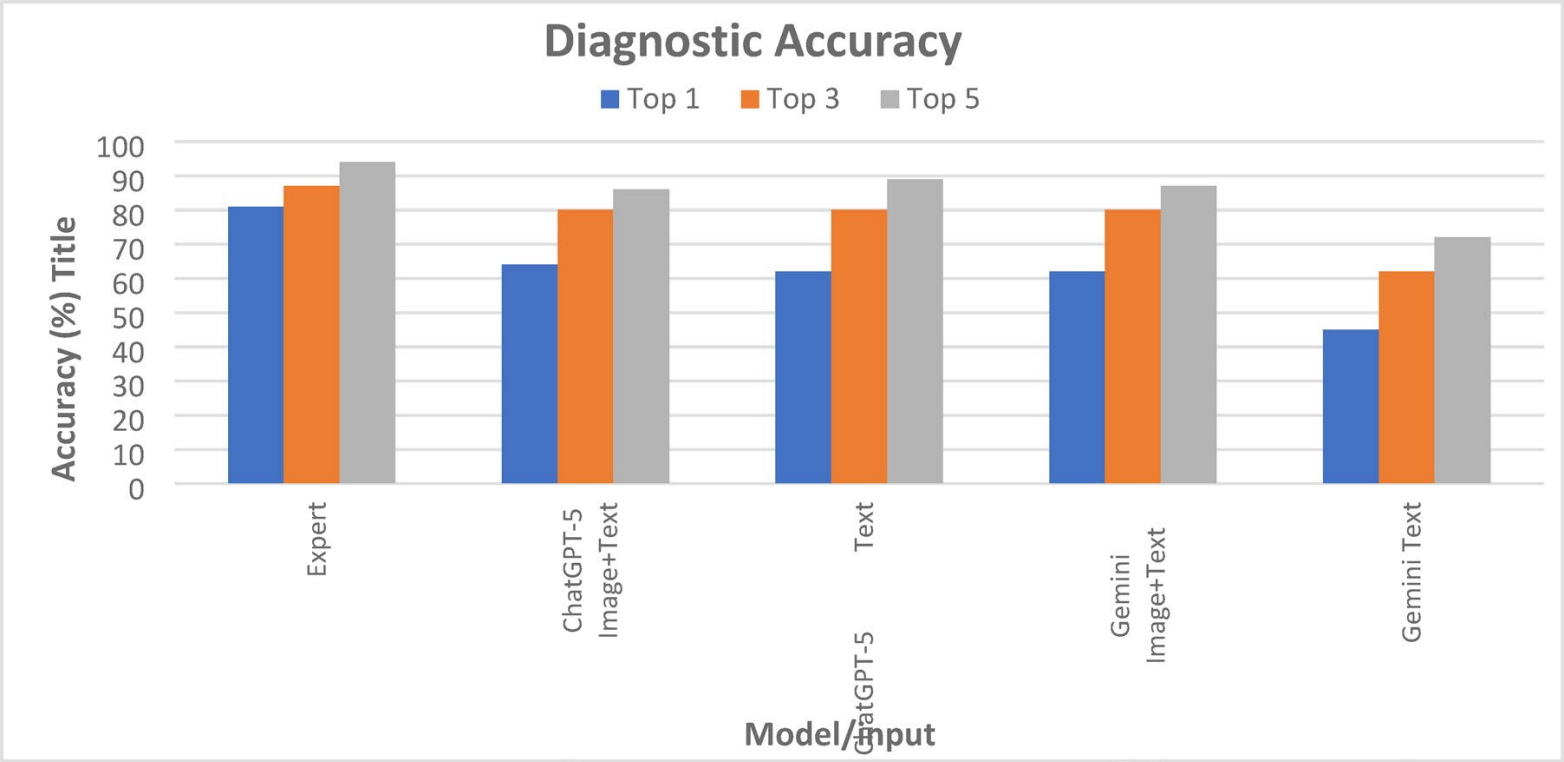
Bar Chart showing Diagnostic Accuracy (%) for ChatGPT-5, Gemini 2.5 Pro, and Expert across different thresholds (Top 1,Top 3, Top 5) for both ‘Text + Images’ and ‘Text Only’ conditions.

Cochran’s Q tests confirmed significant overall differences among AI conditions at all thresholds (Top-1: χ^2^ = 37.4, Top-3: χ^2^ = 29.4, Top-5: χ^2^ = 24.8; all *p* < 0.001). Expert performance was consistently highest, achieving 81.0%, 87.0%, and 94.0% at Top-1, Top-3, and Top-5, respectively (Table 1; Fig. 2). At Top-1, all AI conditions were significantly lower than the Expert (p_adj < 0.001), with ChatGPT-5 text-only (45.0%) performing markedly worse than the other three AI conditions (62.0–64.0%), which did not differ from one another. At Top-3, the Expert remained superior to ChatGPT-5 text-only (62.0%; p_adj < 0.001) but was not significantly different from ChatGPT-5 image + text or either Gemini condition (all 80.0%); within the AI group, ChatGPT-5 text-only was significantly lower, while the other three were equivalent. At Top-5, the Expert (94.0%) was again significantly higher than ChatGPT-5 text-only (72.0%; p_adj < 0.001) but comparable to ChatGPT-5 image + text (86.0%), Gemini image + text (87.0%), and Gemini text-only (89.0%). Overall, the Expert consistently performed best, ChatGPT-5 text-only consistently performed worst, and the addition of visual input substantially improved ChatGPT-5 accuracy, bringing it to Gemini levels and, at broader ranks, approximating the Expert (Table 1; Fig. 2).

**Table 1:** Rank-based diagnostic accuracy with Cochran’s Q and pairwise groupings.

Rank	Expert	ChatGPT-5		Gemini 2.5 Pro		Cochran Q χ^2^ (df = 3)	*p*-value
		Image + text	Text	Image + text	Text		
Top 1	162 (81.0%)^a^	128 (64.0%)^b^	124 (62.0%)^b^	124 (62.0%)^b^	90 (45.0%)^c^	37.4	< 0.001
Top 3	174 (87.0%)^a^	160 (80.0%)^b^	160 (80.0%)^b^	160 (80.0%)^b^	124 (62.0%)^c^	29.4	< 0.001
Top 5	188 (94.0%)^a^	172 (86.0%)^ab^	178 (89.0%)^ab^	174 (87.0%)^ab^	144 (72.0%)^c^	24.8	< 0.001

^a^: Statistically equivalent to expert (p_adj ≥ 0.05). ^b^: Statistically equivalent to each other, but significantly lower than expert (p_adj < 0.05 vs. expert). ^c^: Significantly worse than all other groups (p_adj < 0.05). Cochran’s Q was applied to the four AI conditions only (df = 3). The Expert column is presented for reference and was not included in statistical tests.

### Vision-weighted diagnostic gain (VWDG) comparison

**Fig. 3:**
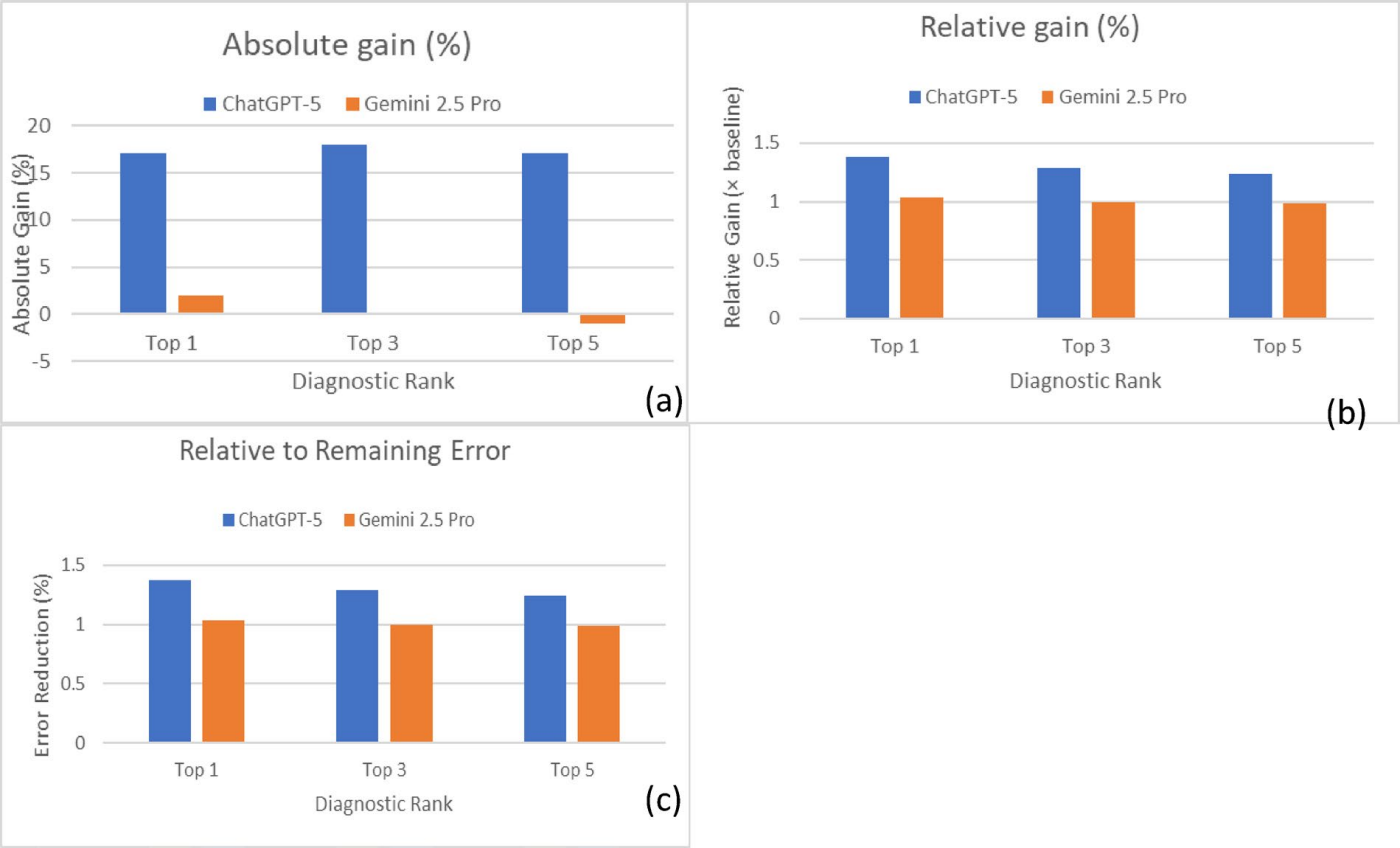
Vision-Based Diagnostic Gain (VWDG) of ChatGPT-5 and Gemini 2.5 Pro across diagnostic ranks. (**a**) Absolute gain (percentage points), (**b**) relative gain (× baseline), and (**c**) gain relative to remaining error (%). ChatGPT-5 consistently outperformed Gemini across all ranks.

The Vision-Based Diagnostic Gain (VWDG) analysis showed that ChatGPT-5 derived substantial benefit from visual inputs, whereas Gemini exhibited minimal or even negative changes. At Top-1, ChatGPT-5 improved by + 17% points (relative gain ×1.38; error reduction 30.9%), compared with a negligible + 2-point gain for Gemini (×1.03; error reduction 5.3%). The absolute advantage of ChatGPT-5 over Gemini was + 15 points (Cohen’s h = 0.34; small-to-medium effect; *p* < 0.001). At Top-3, ChatGPT-5 improved by + 18 points (×1.29; error reduction 47.4%), while Gemini showed no change. The differential gain was + 18 points (Cohen’s h = 0.41; medium effect; *p* < 0.001). At Top-5, ChatGPT-5 again improved by + 17 points (× 1.24; error reduction 60.7%), whereas Gemini slightly declined (− 1 point; ×0.99; error reduction − 7.7%). The differential gain was + 18 points (Cohen’s h = 0.39; small-to-medium effect; *p* < 0.001). Overall, visual information significantly enhanced ChatGPT-5’s performance across all ranks, producing small-to-medium effect sizes, while Gemini’s performance remained essentially unchanged. These results highlight that ChatGPT-5 is strongly vision-dependent for diagnostic accuracy, whereas Gemini is largely vision-stable (Table 2; Fig. 3).

**Table 2:** Vision-based diagnostic gain (VWDG) metrics comparing ChatGPT-5 and Gemini 2.5 Pro across diagnostic ranks (*N* = 200 cases).

Rank	VWDG metric	ChatGPT-5	Gemini 2.5 Pro	Δ (ChatGPT-5 – Gemini 2.5 Pro)	*P*-value†	Cohen’s h‡
Top-1	Absolute gain (pp)	+ 17.0	+ 2.0	+ 15.0	< 0.001	0.34
	Relative gain to baseline	×1.38	×1.03	+ 0.35	< 0.001	
	Gain vs. remaining error	30.9%	5.3%	+ 25.6%	< 0.001	
Top-3	Absolute gain (pp)	+ 18.0	0.0	+ 18.0	< 0.001	0.41
	Relative gain to baseline	×1.29	×1.00	+ 0.29	< 0.001	
	Gain vs. remaining error	47.4%	0.0%	+ 47.4%	< 0.001	
Top-5	Absolute gain (pp)	+ 17.0	−1.0	+ 18.0	< 0.001	0.39
	Relative gain to baseline	× 1.24	×0.99	+ 0.25	< 0.001	
	Gain vs. remaining error	60.7%	− 7.7%	+ 68.4%	< 0.001	

† Absolute-gain *P*-values use a Wald test on the difference-of-differences in proportions. Relative-gain *P*-values compare the models’ log risk ratios (delta-method variance). § For “gain vs. remaining error” no closed-form variance is standard; the same *P* as the absolute-gain test is provided as a conservative approximation. ‡ Cohen’s *h* is the arcsine-transformed difference between ChatGPT-5 and Gemini 2.5 Pro for the given VWDG metric. Magnitude labels follow the common benchmarks (trivial < 0.2 < small < 0.5 < medium < 0.8 < large).

### Impact of combining images based on lesion type diagnosis

Between-model comparisons showed that Gemini consistently outperformed ChatGPT-5 in text-only conditions, particularly for malignant and inflammatory lesions. At Top-1, Gemini text-only reached 65.0% compared with 30.0% for ChatGPT-5 (Δ − 35.0 pp; *p* < 0.001; h = 0.74; large effect). Similar but smaller gaps were observed at Top-3 and Top-5 for malignant lesions (Δ − 25.0 pp; *p* = 0.012 and 0.003, respectively; h ≈ 0.39–0.50; small-to-medium to medium effects). However, when images were added, ChatGPT-5 achieved gains of + 20–26 pp across malignant, reactive, and inflammatory lesions, narrowing or reversing the gap with Gemini. For example, at Top-3 reactive lesions, ChatGPT-5 Image + Text reached 85.7% compared with 74.3% for Gemini (Δ + 11.4 pp; *p* < 0.001; h = 0.38; small-to-medium effect). At Top-5 inflammatory lesions, ChatGPT-5 achieved 94.1% versus 91.2% for Gemini (Δ + 2.9 pp; *p* < 0.001; h = 0.45; small-to-medium effect). Benign lesions showed moderate accuracy but no vision-based benefit in either model, with no significant differences across ranks (all *p* ≥ 0.5; h ≤ 0.18; trivial-to-small effects). Gemini was stronger in text-only settings, but ChatGPT-5 demonstrated much larger vision-based gains, enabling it to match or surpass Gemini once images were incorporated—especially for malignant, reactive, and inflammatory lesions (Table 3).

**Table 3:** Lesion-type–stratified diagnostic performance of ChatGPT-5 and Gemini 2.5 Pro with and without visual input across Top-1, Top-3, and Top-5 ranks (N = 200 cases).

Rank	Lesion type (*n*)	ChatGPT-5 Img/Text (% gain)	Gemini Img/Text (% gain)	Δ Img (ChatGPT-5 – Gemini)	Δ Text (ChatGPT-5 – Gemini)	*p* value†	Cohen’s h‡
Top-1	Malignant (40)	55.0 / 30.0 (+ 25.0)	70.0 / 65.0 (+ 5.0)	− 15.0	− 35.0	0.004	0.74
	Benign (22)	36.4 / 36.4 (0.0)	45.5 / 54.5 (− 9.1)	− 9.1	− 18.1	0.688	0.15
	Reactive (70)	62.9 / 54.3 (+ 8.6)	62.9 / 48.6 (+ 14.3)	0.0	+ 5.7	0.078	0.11
	Inflammatory (68)	73.5 / 47.1 (+ 26.5)	67.6 / 73.5 (− 5.9)	+ 5.9	− 26.4	< 0.001	0.55
Top-3	Malignant (40)	70.0 / 50.0 (+ 20.0)	80.0 / 75.0 (+ 5.0)	−10.0	− 25.0	0.012	0.50
	Benign (22)	54.5 / 54.5 (0.0)	63.6 / 72.7 (− 9.1)	− 9.1	− 18.2	0.500	0.18
	Reactive (70)	85.7 / 68.6 (+ 17.1)	74.3 / 71.4 (+ 2.9)	+ 11.4	− 2.8	< 0.001	0.38
	Inflammatory (68)	88.2 / 67.6 (+ 20.6)	91.2 / 88.2 (+ 2.9)	− 3.0	− 20.6	0.004	0.42
Top-5	Malignant (40)	85.0 / 60.0 (+ 25.0)	90.0 / 85.0 (+ 5.0)	− 5.0	− 25.0	0.003	0.39
	Benign (22)	54.5 / 54.5 (0.0)	72.7 / 72.7 (0.0)	− 18.2	− 18.2	1.000	0.18
	Reactive (70)	94.3 / 74.3 (+ 20.0)	85.7 / 82.9 (+ 2.9)	+ 8.6	− 8.6	< 0.001	0.36
	Inflammatory (68)	94.1 / 70.6 (+ 23.5)	91.2 / 91.2 (0.0)	+ 2.9	− 20.6	< 0.001	0.45

†*p* values are from McNemar tests comparing paired predictions. ‡ Cohen’s h effect size between ChatGPT-5 and Gemini (0.20 small, 0.50 medium, 0.80 large). Gains in parentheses = absolute vision-based gain (percentage points).

### Impact of combining images based on difficulty cases diagnosis

When stratified by case difficulty, Gemini again outperformed ChatGPT-5 in text-only mode, particularly in high-difficulty cases. For example, at Top-1 high-difficulty, Gemini text-only reached 68.6% compared with 42.9% for ChatGPT-5 (Δ − 25.7 pp; *p* = 0.077; Cohen’s h = 0.52; medium effect). However, ChatGPT-5 achieved substantial vision-based gains in moderate-difficulty cases, improving by + 30 pp at Top-1, + 20 pp at Top-3, and + 15 pp at Top-5 (all *p* < 0.001). These gains enabled ChatGPT-5 to converge with or surpass Gemini when images were included. In low-difficulty cases, both models performed well, and differences between them were small and not statistically significant (Top-1: *p* = 0.754; Top-3: *p* = 0.077; Top-5: *p* = 0.125; all effects small-to-medium). In summary, Gemini showed stronger robustness in text-only predictions, especially in high-difficulty cases. However, ChatGPT-5 derived much larger benefits from visual inputs, particularly in moderate-difficulty cases, where vision-based gains closed or reversed the performance gap with Gemini (Table 4).

**Table 4:** Vision-based diagnostic gain (Text + Images vs. Text-only) and between-model comparisons for ChatGPT-5 and Gemini 2.5 Pro, stratified by lesion type and case difficulty (*N*  = 200).

Rank	Difficulty (*n*)	ChatGPT-5 Img/Text (% gain)	Gemini Img/Text (% gain)	Δ Img (pp)	Δ Text (pp)	*p* value†	Cohen’s h‡
Top-1	Low (50)	44.0 / 40.0 (+ 4.0)	60.0 / 56.0 (+ 4.0)	− 16.0	− 16.0	0.754	− 0.32
	Middle (80)	80.0 / 50.0 (+ 30.0)	62.5 / 60.0 (+ 2.5)	+ 17.5	− 10.0	< 0.001	0.39
	High (70)	54.3 / 42.9 (+ 11.4)	68.6 / 68.6 (0.0)	−14.3	− 25.7	0.077	− 0.52
Top-3	Low (50)	72.0 / 56.0 (+ 16.0)	80.0 / 80.0 (0.0)	−8.0	− 24.0	0.077	− 0.49
	Middle (80)	90.0 / 70.0 (+ 20.0)	82.5 / 77.5 (+ 5.0)	+ 7.5	− 7.5	< 0.001	0.22
	High (70)	65.7 / 54.3 (+ 11.4)	71.4 / 74.3 (− 2.9)	−5.7	− 20.0	0.125	−0.43
Top-5	Low (50)	80.0 / 64.0 (+ 16.0)	84.0 / 84.0 (0.0)	−4.0	− 20.0	0.125	−0.44
	Middle (80)	95.0 / 80.0 (+ 15.0)	92.5 / 87.5 (+ 5.0)	+ 2.5	− 7.5	< 0.001	0.17
	High (70)	74.3 / 65.7 (+ 8.6)	82.9 / 82.9 (0.0)	−8.6	− 17.2	0.250	− 0.36

† *p* values from McNemar tests (exact test when discordant ≤ 25). ‡ Cohen’s h effect size: 0.20 small, 0.50 medium, 0.80 large. Gains in parentheses = within-model vision-based improvement (percentage points).

### Multivariable regression analysis

Multivariable within-case analyses showed that the addition of clinical photographs and radiographs conferred a marked and independent diagnostic benefit for ChatGPT-5, but not for Gemini 2.5 Pro. Across all 200 cases, ChatGPT-5 achieved odds ratios of 5.25 at Top-1, 4.60 at Top-3, and 6.67 at Top-5 for images versus text (all *p* < 0.001), indicating a several-fold increase in the likelihood of correct diagnosis when visual information was incorporated. In contrast, Gemini’s ORs were close to unity and non-significant (all *p* ≥ 0.6), underscoring its relative insensitivity to image input. When stratified by lesion type, the effect of images on ChatGPT-5 was strongest in inflammatory and reactive lesions. Inflammatory cases showed very large improvements, with OR = 37.0 at Top-1 (*p* < 0.001) and OR = 9.0 at Top-5 (95% CI 2.09–38.79; *p* < 0.001). Reactive lesions also benefited greatly (Top-3: OR = 25.0, *p* < 0.001; Top-5: OR = 29.0, *p* < 0.001). Malignant lesions demonstrated significant incremental benefit at Top-1 (OR = 6.0, *p* = 0.013) and Top-3 (OR = 6.0, *p* = 0.013), but not at Top-5 (OR = 9.0, *p* = 0.125). Benign lesions showed no image effect (OR ≈ 1.0 across ranks, all *p* = 1.000). For Gemini, no lesion subgroup yielded a significant benefit, and some effects were inconsistent in direction, including ORs below unity.

When stratified by case difficulty, ChatGPT-5 derived the greatest incremental benefit from images in moderately difficult cases, with ORs of 49.0 at Top-1, 45.0 at Top-3, and 41.0 at Top-5 (all *p* < 0.001). Low-difficulty cases showed smaller and non-significant improvements (OR = 1.5–3.5, *p* ≥ 0.18). High-difficulty cases yielded borderline or non-significant gains (Top-1: OR = 3.0, *p* = 0.077; Top-3: OR = 2.33, *p* = 0.115), and at Top-5 Gemini showed a significant negative effect (OR = 0.08, *p* = 0.031). Tn summary, visual information independently and substantially boosted ChatGPT-5’s diagnostic accuracy, particularly in inflammatory and reactive lesions and in moderately difficult cases. Malignant lesions showed gains at Top-1 and Top-3 but not at Top-5, while benign lesions remained unaffected. Gemini’s performance was essentially unchanged, and in some high-difficulty cases visual input even reduced accuracy, highlighting a fundamental difference in how the two models integrate visual data (Table 5).

**Table 5:** Independent and incremental effects of clinical photographs/radiographs on ChatGPT-5 and Gemini 2.5 Pro diagnostic accuracy across lesion types and difficulty levels (paired logistic analysis, n = 200).

Rank	Stratum	ChatGPT-5 OR	95% CI	*p*	Gemini 2.5 Pro OR	95% CI	*p*
Top-1	Images present (all 200)	5.25	2.46–11.18	< 0.001	1.18	0.67–2.09	0.665
	Malignant	6.00	1.34–26.81	0.013	1.00	0.32–3.10	1.000
	Benign	1.00	0.25–4.00	1.000	0.50	0.09–2.73	0.688
	Reactive	4.00	0.85–18.84	0.109	2.25	0.98–5.17	0.078
	Inflammatory	37.00	2.23–613.99	< 0.001	0.11	0.01–2.06	0.125
	Low difficulty	1.50	0.42–5.32	0.754	1.25	0.49–3.17	0.815
	Moderate difficulty	49.00	2.98–805.79	< 0.001	1.50	0.42–5.32	0.754
	High difficulty	3.00	0.97–9.30	0.077	1.00	0.42–2.40	1.000
Top-3	Images present (all 200)	4.60	2.32–9.12	< 0.001	1.00	0.45–2.23	1.000
	Malignant	6.00	1.34–26.81	0.013	0.50	0.09–2.73	0.688
	Benign	1.00	0.25–4.00	1.000	0.20	0.01–4.17	0.500
	Reactive	25.00	1.48–422.26	< 0.001	1.33	0.46–3.84	0.791
	Inflammatory	4.50	1.52–13.30	0.004	5.00	0.24–104.15	0.500
	Low difficulty	2.50	0.78–7.97	0.180	1.50	0.42–5.32	0.754
	Moderate difficulty	45.00	2.73–741.85	< 0.001	2.00	0.37–10.92	0.688
	High difficulty	2.33	0.90–6.07	0.115	0.33	0.07–1.65	0.289
Top-5	Images present (all 200)	6.67	2.83–15.72	< 0.001	0.75	0.26–2.16	0.791
	Malignant	9.00	0.48–167.17	0.125	0.11	0.01–2.06	0.125
	Benign	1.00	0.25–4.00	1.000	1.00	0.02–50.40	1.000
	Reactive	29.00	1.73–486.16	< 0.001	1.50	0.42–5.32	0.754
	Inflammatory	9.00	2.09–38.79	< 0.001	1.00	0.02–50.40	1.000
	Low difficulty	3.50	1.15–10.63	0.031	3.00	0.61–14.86	0.289
	Moderate difficulty	41.00	2.48–677.92	< 0.001	1.00	0.02–50.40	1.000
	High difficulty	3.00	0.61–14.86	0.289	0.08	0.00–1.37	0.031

ORs are within-case odds ratios (images vs. text) from discordant-pair analysis (Haldane–Anscombe corrected if needed).

95% CIs are Wald intervals; p values from McNemar’s test (exact if discordant ≤ 25).

*indicate (*p* < 0.05) indicate statistically significant incremental gain from images.

## Discussion

In comparison to knowledgeable oral medicine specialists, the current study offers a thorough, practical assessment of the diagnostic abilities of two cutting-edge multimodal AI models, ChatGPT-5 and Gemini 2.5 Pro. Our results show a complicated and dynamic environment where model architecture, input modality (text vs. text + image), lesion type, and case difficulty all have a significant impact on AI performance. Importantly, these findings demonstrate the quick speed of progress in AI diagnostics and represent a substantial divergence from our earlier work with earlier model iterations^19,23^.

To the best of our knowledge, VWDG has not been previously defined or systematically used in clinical AI research. This study is, therefore, the first to quantify the incremental diagnostic value that visual prompting brings to multimodal large language models in a real-world, expert-anchored setting.

At the Top-3 and Top-5 ranks, and even in text-only mode for Gemini, our most remarkable discovery is that ChatGPT-5 with visual input achieved diagnostic accuracy statistically equivalent to human experts. This is a significant advancement. Even though AI models demonstrated promise in an earlier study^23^, human experts continued to have a considerable advantage, especially at the demanding Top-1 level. According to the current findings, the most recent generation of vision-language models has started to bridge this gap by improving their capacity to integrate and rationalize with multimodal clinical data fundamentally rather than just slightly.

This observation aligns with a growing body of evidence from various medical specialties. While research in dermatology has repeatedly demonstrated that AI can match dermatologists in image-based classification tasks^11^, a recent study in orthopedics found that GPT-4 approached expert-level performance in generating differential diagnoses from clinical vignettes^24^. By proving this capability in the intricate and visually subtle field of oral medicine, our study adds to this consensus.

The expert’s consistent superiority in Top-1 accuracy (81.0% vs. 62.0–64.0% for multimodal AI) highlights the fact that the first, conclusive diagnostic step, the most important for patient anxiety and initial management, still depends on complex clinical knowledge that AI has not yet completely grasped. This subtlety is frequently overlooked in more general accuracy metrics. It is consistent with the findings of Hirosawa et al. (2023), who observed that although ChatGPT-generated differential lists were accurate, complex internal medicine cases did not always have the best diagnosis^25^.

The measurement of Vision-Based Diagnostic Gain (VWDG) is a key and innovative contribution of this work. To ensure a fair yet clinically realistic comparison between AI models and human experts, differences in input modality were carefully structured according to the study’s primary objective. Although human experts were provided only with multimodal case information (text and images), this design was deliberate to preserve clinical realism, as visual examination is integral to oral diagnostic reasoning. In contrast, ChatGPT-5 and Gemini 2.5 Pro were evaluated under both *text-only* and *text + image* conditions to isolate the specific contribution of visual data, expressed as the Vision-Based Diagnostic Gain (VBDG). This approach ensured fairness in benchmarking by allowing direct comparison of diagnostic content while maintaining ecological validity for human interpretation and analytical transparency for model performance.

The findings reveal a key strategic distinction between the two AI models: ChatGPT-5 Depends on Vision across all diagnostic ranks, it obtained a significant, statistically significant benefit from visual inputs (e.g., + 17 to + 18% points in absolute gain). This implies that visual context significantly improves its diagnostic reasoning, enabling it to get around restrictions on its text-only performance. This is a significant and clinically significant improvement, as evidenced by the large effect sizes (Cohen’s h ~ 0.34–0.41).

Vision-Stable Gemini 2.5 Pro: The addition of images had little effect on its performance, and occasionally made it slightly worse. This suggests that while Gemini’s strength is strong textual reasoning, likely acquired from a large body of medical literature, it integrates visual information less successfully. This implies that, in contrast to research that emphasizes the critical role of images, for some models and use cases, thorough textual descriptions might be adequate^26^.

From a clinical perspective, this dichotomy has significant implications. ChatGPT-5 would be most useful as a true multimodal consultant in situations where high-quality clinical images are available. On the other hand, Gemini may be more dependable when used in telemedicine or for initial consultations that mostly rely on textual case descriptions.

Our stratified analysis further explains why human experts maintained a Top-1 advantage. In text-only mode, Gemini consistently outperformed ChatGPT-5 particularly for inflammatory and malignant lesions (e.g., Δ − 35.0 pp for malignant Top-1). pattern also noted by Tomo et al. (2024) across oral and maxillofacial pathologies^27^. The introduction of visual data, however, was a “great equalizer” for ChatGPT-5. It successfully eliminated Gemini’s text-based advantage and frequently enabled it to outperform Gemini’s multimodal performance by achieving enormous vision-based gains (+ 20 to + 26 pp) in malignant, reactive, and inflammatory lesions. A significant strength is its capacity to use visual cues for challenging lesions, which is consistent with research in dermatology AI, where models are highly effective at spotting visual patterns suggestive of cancer^11^.

On the other hand, neither model demonstrated any discernible visual advantage for benign lesions. This is consistent with clinical intuition: benign lesions, such as fibromas and papillomas, frequently present as classic textual lesions with non-distinctive visual features, which reduces the contribution of detailed visual analysis. In this field, the ability of a qualified clinician to recognize patterns based on years of experience is still crucial and challenging for AI to imitate. This result is in direct agreement with earlier findings^23^, highlighting expert superiority in identifying reactive and benign conditions.

According to this “AI difficulty paradox”, AI models may be overfitting to the intricate, pattern-rich data found in their training sets while having trouble making the small, obvious distinctions needed for simple diagnoses. An AI might overthink it by taking into account rarer, more serious differentials, whereas a human expert can quickly identify a classic traumatic ulcer or geographic tongue. This emphasizes AI’s function as a potent decision-support tool, not a substitute, for complicated, ambiguous situations where even human experts might want a second opinion. Work in other domains supports this idea, which best positions AI as an auxiliary tool rather than a self-sufficient diagnostician^28^.

The main goal of the comparison between ChatGPT-5 and Gemini 2.5 Pro is to map out their unique and complementary strengths rather than declare a winner. With its exceptional diagnostic accuracy based solely on textual descriptions, Gemini serves as a highly skilled text-based consultant. However, ChatGPT-5 functions as a true multimodal partner, and it is only when visual data is added that its performance reaches expert level. This contradiction shows that model performance is intrinsically context-defined and not absolute. Selecting the appropriate AI for the task at hand, taking into account the various input modalities, is more important for successful deployment than selecting the “best” AI.

From a translational standpoint, the clinical context must guide AI deployment. Suboptimal results may occur if a text-strong model such as Gemini is used in an image-rich environment, or a vision-dependent model such as ChatGPT-5 is used without image input. In practice, this supports a tiered diagnostic model in which text-based systems perform initial triage, while vision-enabled models handle complex or image-intensive cases. Such tools can enhance specialist augmentation and education, helping clinicians and trainees understand how diagnostic reasoning changes when visual cues are introduced.

### **Strengths and limitations**

This study’s strengths include the use of real-world, histopathologically verified cases and a direct comparison with experienced oral medicine experts. Limitations include a fixed prompting strategy, a single-country dataset, and the inherent pace of model iteration. Continuous validation and multicenter trials will be essential to confirm generalizability and optimize clinical integration. Future work should explore dynamic clinician–AI collaboration and mechanisms for safely incorporating AI outputs into routine oral diagnostic workflows to improve patient outcomes.

## Conclusion

Our analysis of ChatGPT-5 and Gemini 2.5 Pro demonstrates that state-of-the-art multimodal AI models are no longer confined to the role of digital assistants but can function as reliable peers to human experts in differential diagnosis particularly when visual information is incorporated. By introducing and applying VWDG, we quantified the incremental diagnostic value conferred by images beyond text alone. This revealed a strategic divergence: ChatGPT-5 acts as a *visual synergist*, achieving substantial VWDG, whereas Gemini 2.5 Pro performs as a *textual expert* with limited incremental visual benefit. Rather than framing this as a simple contest between models, these findings support a context-aware framework for clinical AI use, where the most appropriate system depends on the diagnostic inputs available. Ultimately, the paradigm is not competition but cooperative synergy: AI provides rapid, multimodal pattern recognition especially valuable in complex cases while human experts contribute nuanced clinical judgment, particularly for straightforward or benign presentations. Harnessing both perspectives, guided by metrics such as VWDG, offers a pathway toward accessible, efficient, and accurate diagnosis in oral medicine.

## Data Availability

Research data supporting this publication is available from the corresponding author upon request.
